# *Vaccinium macrocarpon* (Cranberry)-Based Dietary Supplements: Variation in Mass Uniformity, Proanthocyanidin Dosage and Anthocyanin Profile Demonstrates Quality Control Standard Needed

**DOI:** 10.3390/nu12040992

**Published:** 2020-04-03

**Authors:** Giuseppe Mannino, Vita Di Stefano, Antonino Lauria, Rosa Pitonzo, Carla Gentile

**Affiliations:** 1 Department of Life Sciences and Systems Biology, Innovation Centre, Plant Physiology Unit, University of Turin, Via Quarello 15/A, 10135 Turin, Italy; giuseppe.mannino@unito.it; 2 Department of Biological, Chemical and Pharmaceutical Sciences and Technologies (STEBICEF), University of Palermo, Viale delle Scienze, 90128 Palermo, Italy; antonino.lauria@unipa.it (A.L.); carla.gentile@unipa.it (C.G.); 3 Advanced Technologies Network Center (ATeN Center), University of Palermo, Viale delle Scienze, Ed. 18, 90128 Palermo, Italy; rosa.pitonzo@unipa.it

**Keywords:** American cranberry, HPLC-UV/Vis, Orbitrap, Anthocyanins, Proanthocyanidins, chemical fingerprinting, dietary supplements, *Vaccinium macrocarpon*, BL-DMAC

## Abstract

*Vaccinium macrocarpon* (syn. American Cranberry) is employed in dietary supplements (DS) with the aim to improve urinary tract well-being. This property is linked to the antiadhesion-activity of proanthocyanidins (PACs) against uropathogenic-bacteria. However, the current European legislation has been criticized for being weak and ineffective. Indeed, recent scientific works report mislabeled, contaminated, and adulterated supplements containing dangerous or unknown compounds, or sold at toxic doses. In this work, we analysed 24 DS that claim to contain cranberry, and to have a specific dosage of PACs. Our tests included the control of the good manufacturing practice according to the European Pharmacopoeia, and the verification of the claimed dosage of PACs. Moreover, in order to confirm the real presence of cranberry in DS, chemical fingerprinting via HPLC-UV/Vis-MS/MS was employed. Our results showed that 17 DS did not comply with the uniformity test of dosage forms, and only five contained cranberry. Finally, 16 DS claimed an incorrect amount of PACs. These data suggest that several cranberry-based DS are present in the European market with insufficient quality controls. Considering that often DS are self-prescribed by consumer relying on their claim, the data obtained in this work should encourage more controls and stricter rules.

## 1. Introduction

In recent years, an ever-increasing demand for supplements based on natural products has been recorded in Europe [1], and currently their sales are close to 7 billion Euros annually [2]. In particular, Italy ranks as the leading country in Europe for the consumption of plant-based dietary supplements (DS) [3]. These products are not simply used to support sport performances [4], but their non-prescribed use is very frequent also among children and adolescents [5], students [6], pregnant and postmenopausal women [7,8], and oncological patients or those affected by other chronic diseases [9,10].

However, the current regulation and monitoring systems of DS has been criticized for being weak and ineffective [11]. Indeed, the requirements for the quality control of food and drugs are not mandatory for DS [12]. On one hand, before the registration and introduction into the European market, medicines have to be subjected to scientific evaluation by the European Medicines Agency (EMA), which involves clinical trials to probe their safety, quality, and efficacy [13]. On the other hand, the food safety is controlled by the European Commission, which established a legal framework to regulate the market of food, fortified food, and for so-called functional foods [14]. Moreover, according to the legislation, information about food derivation and origin must be unblemished and unquestionable for the consumer [15].

In this context, food supplements are considered as food, thus their registration, clinical trials, effectiveness, and toxicity testing are not required as they are for medicines or synthetic drugs [16]. However, food supplements are designed to look like synthetic drugs, since they have similar unit-doses and dosage [17]. Moreover, they are sold in pharmacies and, as with medicines, inadvertent overdosing may occur if the recommended posology and dosage are not strictly followed [17].

This borderline situation follows in the marketability of some DS that fail to declare all their active substances [18], the plant parts used for their formulation [19], or the professed amount of active substances [19,20] on their label. Additionally, in several cases a misidentification of the initial plant raw material, or the adulteration with other plants [21] is also found. Furthermore, in some cases, contaminations from environmental chemicals, including heavy metals, pesticide and herbicide residues [22], and mycotoxins and harmful micro-organisms [23,24] have also been reported. Since consumers perceive food supplements as natural, and consequently safe [25], the investigation of their quality and security is very important before their placing on the market.

In this study, we focused our attention on the quality control of 24 European bestselling *Vaccinium macrocarpon*-based DS. All these products share the claim to produce beneficial effects for the well-being of the urinary tract. Indeed, it has been demonstrated that a specific dosage of Cranberry’s proanthocyanidins (PACs), quantified via BL-DMAC assay (Brunswick Laboratories 4-dimethylaminocinnamaldehyde), is sufficient to explicate a significant beneficial effect linked to its antiadhesion activity against uropathogenic P-fimbriated *Escherichia coli* bacteria [26].

In order to evaluate the proper formulation and dosage of the unit doses, mass uniformity, dosage uniformity, and PAC content were evaluated. Finally, in order to properly identify the used raw material, a chemical fingerprinting of all products was performed. 

## 2. Materials and Methods 

The 24 analysed DS comprise the bestselling products, according to the databases reporting the sell-in of medicine, pharmaceutical, and botanical products, including Cranberry-based DS.

### 2.1. European Pharmacopoeia Test for Mass Uniformity

The mass uniformity of the individual unit doses contained in each DS package was done following the European Pharmacopeia directives [27]. Briefly, at least twenty unit doses were taken, and each of them was individually weighted. Then, the average mass and its standard deviation was calculated. The unit doses complied with the test if not more than one individual mass was outside the 5% (tablets) or 7.5% (capsules and sachets) of the average mass. Moreover, tablets and capsules failed to comply with the test if one individual mass was outside the limits of 90%–110% (tablets) or 85%–115% (capsules and sachets) of the average mass.

### 2.2. Extract Preparation and Evaluation of Total Proanthocyanidin Content (tPAC)

For tPAC evaluation, different extract types were prepared depending on the pharmaceutical form of each DS unit dose. In particular, tablets were pulverized and then extracted with 30 mL of PAC Extraction Buffer [75% (*v*/*v*) Acetone with 0.5% (*v*/*v*) Acetic Acid] [28]. If present, the coating was manually removed from tablets before the extraction. Capsules were simply opened, and their total content was sampled and extracted with 20 mL of PAC Extraction Buffer. Sachets were opened and their whole content was extracted with 40 mL of PAC Extraction Buffer. Finally, liquids were directly diluted in 30 mL of PAC Extraction Buffer. All the extracts were vortexed for 5 min, and then placed into an ultrasound bath at room temperature for 30 min. The solution was further mixed on a swing plate for 1 h. Samples were then centrifuged at 5000× *g* for 5 min, and the supernatant was collected and used for the quantification of tPAC via BL-DMAC assay [28]. In order to evaluate the completeness of the extraction process, the residues were also assayed. Extractions and quantifications were performed in triplicate.

### 2.3. Extracts Preparation and Evaluation of Total Anthocyanins Content (tTAC)

For tTAC evaluation, different type of extracts were prepared depending on the pharmaceutical form of each DS unit dose. In particular, tablets were pulverized and extracted with 20 mL of TAC Extraction Buffer [50% (*v*/*v*) Methanol with 1% (*v*/*v*) of Formic Acid] [29]. If present, the coating was manually removed from the tablets before extraction. Capsules were simply opened, and their total content was sampled and then extracted with 10 mL of TAC Extraction Buffer. Sachets were opened and the whole content was extracted with 30 mL of TAC Extraction Buffer. Finally, liquids were directly diluted in 10 mL TAC Extraction Buffer.

All the extracts were vortexed for 5 min, and then were placed into an ultrasound bath at room temperature for 30 min. The solution was further mixed on a swing plate for 1h. Samples were centrifuged at 5000× *g* for 10 min, and the supernatant was then collected and used for the quantification of tTAC via pH differential method [29]. Briefly, twenty μL of each extract was added separately to 980 μL of 0.025 M potassium chloride (pH 1.0), or to 980 μL of 0.4 M sodium acetate (pH 4.5). In order to prepare 100 mL of 0.025 M potassium chloride (pH 1.0) buffer, 0.19 g of KCl were weighted, and the solution was adjusted to the final desired pH by using HCl. To prepare the same amount of 0.4 M sodium acetate (pH 4.5) buffer, 5.44 g of CH_3_COONa were weighted, and the solution was adjusted to the final desired pH by using CH_3_COOH or NaOH. The absorbance was measured at 535 nm and 720 nm for both solutions, using 50% (*v*/*v*) Ethanol with 1% (*v*/*v*) formic acid as a blank. The assay was performed in triplicate. The tTAC was calculated using the following equation:

$$tTAC=\frac{\left(\Delta Abs_{pH1}-\Delta Abs_{pH4}\right)\times MW\times DF\times 1000)}{\varepsilon \times l}$$

where: *Δ_Abs_* is the difference in the Abs recorded at 515 nm and 700 nm, at both *pH* 1.00 and *pH* 4.5; *MW* and ε are respectively the molecular weight (449.2 g mol^−1^), and the molar extinction coefficient (26.900 mL mM^−1^ cm^−1^) of cyanidine-3-glucoside, which was used as the reference compound; *DF* is the sample dilution factor; l is the path length (1 cm). The total anthocyanins were expressed as mg of cyanidin-3-glucoside equivalents per dosage unit. In order to evaluate the completeness of the extraction process, the residues were also assayed. Limit of detection (LOD) and Limit of Quantification (LOQ) were calculated using calibration curves of pure cyanidine chloride standard (Extrasynthase^®^, France), ranging between 1–20 μg/mL. LOD and LOQ were calculated as previously reported [30,31].

### 2.4. Chemical Fingerprinting of V. macrocarpon by HPLC-UV-VIS Orbitrap LC-MS detection

In order to assess the raw material used for the formulation of *V. macrocarpon*-based DS, chemical fingerprinting was performed by HPLC-UV/Vis, as previously described by Brown and Shipley [32]. Moreover, in order to identify a possible sophistication, all the DS showing an anthocyanin profile not attributable to *V. macrocarpon* were additionally analysed by UHPLC system, coupled to a quadrupole Orbitrap-mass spectrometer. All the obtained chromatographic profiles were compared to those of authentic *V. macrocarpon* fruits.

#### 2.4.1. HPLC-UV/Vis. HPLC 

The system consisted of an Agilent Technologies 1100, coupled to a UV-VIS detector, and the separation was carried out at constant flow rate (1.3 mL min^−1^) using 10% (*v*/*v*) formic acid (Solvent A) and 50% (*v*/*v*) methanol, acidified with 10% (*v*/*v*) formic acid (Solvent B). The elution method involved a multistep linear solvent gradient: 0–5 min, 10% B; 5–15 min linear increase to 45% B; 15–20 min increase to 60% B, 20–25 linear decrease to 10% B. The total analysis lasted 25 min, with an equilibration time of 5 min. Injected volume of the samples was 20 μL of solution. The column was a reverse phase C18 Luna column (5μm, 150 × 4.6 mm i.d., Phenomenex, USA) maintained at 50 °C by an Agilent 1100 HPLC G1316A Column Compartment. The UV-VIS spectra were registered at 520 nm. The identification of each compound was carried out by the comparison of both retention time and UV-VIS spectra of authentic reference compounds and by using literature data.

#### 2.4.2. Orbitrap LC-MS detection 

A UHPLC (Dionex UltiMate 3000 Rapid Separation LC) system by Thermo Fisher Scientific (San Josè, California) equipped with an autosampler, and controlled by Chromeleon 7.2 software by Thermo Fisher and by Dionex Softron GmbH (Germering, Germany) was used. The UHPLC system was coupled to a quadrupole Orbitrap mass spectrometer (Q Exactive; Thermo Scientific), equipped with an electrospray ion source. The conditions for the detection of anthocyanins were optimized by infusion of a solution of cyanidin-3-O-glucoside and cyanidin-3,5-O-diglucoside in positive-ion mode. The instrumental set-up was under the following conditions: sheath gas flow rate 35 (arbitrary units); auxiliary gas unit flow rate 15 (arbitrary units); spray voltage 3.5 kV; S lens RF 50; capillary temperature 300 °C. Separations were carried out on a UHPLC Luna^®^ column C18(2) 100 Å (150 mm × 2 mm 3 µm, Phenomenex, USA). The injection volume was 1.0 μL. Mobile phase composition: 1% (*v*/*v*) formic acid in water (Solvent A), 1% (*v*/*v*) formic acid in methanol (Solvent B), at a flow rate of 150 μL min^−1^. The gradient was: 0–5 min, 10% B; 5–10 min linear increase to 45% B; 10–15 min hold 45% B; 15–20 min, linear increase to 60% B; 20–25 min, linear increase to 90% B, 25–28 min, hold 90% B, 28–32 min, linear decrease to 10% B, 32–35 min, hold 10% B. The MS detection was conducted in Full MS / dd-MS^2^ (TopN), with positive ion polarity, together with PRM experiment to confirm data obtained. The mass resolution of the Orbitrap analyzer was 70000 and the scan range was 400–700 *m*/*z*. Inclusion mass list is reported in Supplementary Table S1. Mass accuracy was checked daily by injecting a multi-compound standard solution, whereas every 2 days, the analyzer was calibrated with mass accuracy standards. Compound identification was carried out by comparison of both retention time and mass-fragmentation partner of each compound.

### 2.5. Statistical Analysis

Each extract was analysed in triplicate. All results are expressed as mean ± standard deviation (SD). All statistical analyses, tables, and graphs were done using graphpad Prism v5 (GraphPad Software, San Diego, CA USA), SYSTAT version 10.0 (SPSS, Chicago, IL, USA), and Microsoft Excel.

## 3. Results and Discussion

### 3.1. European Pharmacopoeia Test for Mass Uniformity

The main objective of the European Pharmacopeia is to provide the guidelines of pharmaceutical dosage forms through a series of quality control tests. In particular, in order to avoid possible side effects or lack of effect, the uniformity of dosage unit must be satisfied. The term “uniformity of dosage unit” is defined as “the degree of uniformity in the amount of the drug substance among the dosage units” (European Pharmacopeia, 2002). It can be demonstrated by both the evaluation of drug content uniformity or by the evaluation of mass uniformity among the dosage units. Due to sophisticated and expensive instrumentations required for the chemical analytical testes, the mass uniformity is the parameter that can be more easily examined. The weight variation test is based on the comparison of the individual weight of the pharmaceutical forms with an upper and lower percentage limit. In particular, DS packages complied with the test if not more than one individual dosage unit was outside of the percentage established with respect to the average mass (5% for tablets and 7.5% for capsules and sachets) (Criteria 1), but they failed to comply with the test if at least one individual mass was outside of the double of these limits (10% for tablets and 15% for capsules and sachets) (Criteria 2). Mass uniformity results are reported in Table 1.

Among the observed DS, seven of them (#8, #9, #10, #12, #16, #17, and #22) had more than one dosage unit with a mass outside the 5% and 7.5% limit of their average mass. In particular, #8, #9, #10, and #12 had also at least one unit dosage with a weight outside the 10% and 15% range of their average mass. Moreover, some of the DS that did not satisfy Criteria 1 and Criteria 2, had an individual dosage unit mass outside of the 10% of the weight declared in the package label. Therefore, these DS, together with #15 and #17, did not satisfy Criteria 3. In particular, #9, #11, #15, and #17 showed an average mass 15% higher than that declared in the label, while #10 showed an average mass that was about 60% lower.

In conclusion, according to European Pharmacopeia (Criteria 1 and Criteria 2), only 17 of the 24 DS (#1, #2, #3, #4, #5, #6, #7, #11, 13, #14, #15, #18, 19, #20, #21, #23, and #24) satisfied the guidelines of the uniformity of pharmaceutical dosage, but two of them (#11 and #15) claimed, in the label, a weight of individual dosage unit that was largely different from the one empirically evaluated.

### 3.2. Evaluation of tPAC in V. macrocarpon-Based Dietary Supplements

Even though, in the past, the beneficial properties of cranberry was linked to bacteriostatic effect of its acidity [33], recent scientific data showed that this protective effect may depend on the antiadhesion activity of PACs against uropathogenic bacteria [34,35]. In addition, a randomized clinical trial showed that a daily dose of at least 36 mg PACs measured via BL-DMAC assay is necessary to explicate a significative beneficial effect [36]. Consequently, the efficacy of these preparations was strongly related to the amount of PACs in the unit doses determined through this spectrophotometric assay. For this reason, almost all *V. macrocarpon*-based DS on the market, including those examined in this work, claim not only a specific PAC dosage, but also PAC quantification via BL-DMAC. This assay is considered to be the most rapid and accurate spectrophotometric method to determine the total content of PACs, considering all grades of their polymerization [28], and excluding the interference of anthocyanins [37]. Although currently other methods exist for PACs quantification, including those based on liquid chromatographic systems [38], the manufacturer preference to quantify PACs via BL-DMAC assay should not be surprising. Indeed, liquid chromatographic techniques, coupled with mass-spectrometer (LC-MS), may be very expensive, even requiring trained personnel that would increase production costs. Moreover, due to the enormous variability in PAC polymerization-degree, not every LC-MS system is able to carry out an accurate quantification of the total PAC amount [39,40,41].

In this study, we firstly evaluated if the observed DS contained PACs. For this estimation, we decided to perform BL-DMAC assay. On one hand, the results obtained by these quantifications were employed in order to check if the DS that claimed to have a certain PAC amount quantified via BL-DMAC assay actually had the stated dosage. On the other hand, all the DS were analysed by this assay in order to understand if, following the indication of the leaflet, the suggested dosage of 36 mg per day measured via BL-DMAC assay [36], was insured. Considering DS labels, PACs content was claimed in 16 (#1, #3, #4, #5, #6, #7, #8, #9, #10, #14, #16, #17, #18, #19, #20, and #24) of the 24 observed DS. However, only eight of them (#4, #5, #6, #8, #9, #10, #19, and #24) also declared that BL-DMAC was the analytical method used for PACs quantification. However, after the BL-DMAC assay, we revealed that all the observed DS contained PACs, including those that did not report it in the label (Table 2). 

Among the DS that declared PAC content and BL-DMAC as analytical methods for their quantification, our results showed that #4, #5, #19, and #24 contained PACs amount comprised between the 95–105% of the declared value. On the contrary, a content 85% lower than that declared was determined in #9, but a tPAC lower than 15% of that declared was found in #6, #8, and #10. 

Concerning both the supplements that declared PAC content without specifying the analytical method used for their quantification, and those that did not report it, our analysis were limited to the simple estimation of PACs in the unit doses. Moreover, BL-DMAC assay was also performed to evaluate if the suggested posology insured the daily intaking of 36 mg of PAC [36]. Our results showed that #3, #4, #5, #9, #19, #20, #22, and #24 were the only supplements in which the daily intaking of PACs was guaranteed. For other DS, the posology suggested in the label allows the achievement of a daily administration ranged between 0.56 and 12.96 mg of PAC.

### 3.3. Evaluation of TAC in the 24 V. macrocarpon-Based Dietary Supplements

When powder extracts are used for the preparation of plant-based DS, they cannot be used for taxonomic examination to identify their starting raw material. Specific chemical markers instead can be useful to discriminate their plant origin. Cranberry fruits are characterized by a high amount of anthocyanins [42]. Therefore, anthocyanins should always be present in powder extracts that claim to be obtained from *V. macrocarpon* fruits. In order to evaluate TAC, the pH differential method was used. This method makes it possible to selectively determine the real content of anthocyanins in samples, excluding at the same time the contribution of others chemical colorants or red natural pigments [43].

Although our analysis showed that all the observed DS contained PACs (Table 2), five of the 24 *V. macrocarpon*-based DS (#8, #11, #12, #21, and #23) did not contain anthocyanins (LOD: 3 μg mL^−1^; LOQ: 10 μg mL^−1^). For all the other supplements, we recorded a TAC value ranging between 0.389 ± 0.032 to 21.977 ± 1.713 mg of anthocyanin per unit doses. All TAC values are reported in Table 3. 

Considering anthocyanin bioactivity and pharmacological activity, the inclusion of TAC value in the claim of *V. macrocarpon*-based DS would be useful for a correct and complete labelling of these products, even though the current legislation does not foresee it.

### 3.4. Chemical Fingerprinting of the 24 V. macrocarpon-Based Dietary Supplements by HPLC-UV/Vis and Orbitrap LC-MS analysis

Fingerprint techniques, including DNA molecular fingerprint [44] and chemical fingerprinting [45], are crucial for assessing quality of commercial products, and give information about the purity of the original raw material. DNA molecular fingerprinting cannot be applied to the major part of extracts made with organic solvent, due either to the insolubility of nucleic acids in extraction solvents or to DNA instability during manufacture procedures. In these cases, chemical fingerprinting is the best approach, allowing the research of specific chemical markers, which are known to be present in, or also absent from, a particular raw material. Currently, different chemical fingerprinting techniques can be used, with the aim to search for specific markers of the different raw materials. However, all these techniques employ chromatographic systems, but are not always coupled with mass spectrometers. For example, the presence of *V. macrocarpon* fruit in processed products may be checked through the analysis of the general phenolic compositions [46], or focusing on specific bioactive compounds, such as proanthocyanidins [47] or anthocyanins [48,49]. In this context, chemical fingerprinting based on proanthocyanidins or other polyphenols may be less accurate when DS are deliberately adulterated with plants with a similar phytochemical composition. On the other hand, the different anthocyanins have a specific ratio and chemical pattern of distribution among plant species. Consequently, their chemical profile can be useful for better identify the voluntary adulterations of DS [50]. However, the exclusive presence of anthocyanins cannot be considered a sure chemical marker without the identification and characterization of each anthocyanin compound. In particular, even though anthocyanins are one of the largest and most widely spread compounds in several genus of plant, each plant has a specific chemical pattern of distribution [50]. Moreover, although the fruit anthocyanin amount can change due to degradation along processing and storage, the qualitative anthocyanin profile is unique and characteristic [51]. For example, malvidin and pelagordin cannot be present in blackberry fruits [52]. In the same way, delphinidin and malvidin cannot be present in strawberry fruits [53]. On the other hand, *V. macrocarpon* fruits must contain five anthocyanins, for instance cyanidin-3-O-galactoside (C3Ga), cyanidin-3-O-glucoside (C3Gl), cyanidin-3-O-arabinoside (C3Ar), peonidin-3-O-galactoside (P3Ga), and peonidin-3-O-arabinoside (P3Ar) [32,54]. Despite being detected in very low amounts, delphinidin, malvidin, and their related-conjugated cannot be highly present in *V. macrocarpon* fruits [55]. Moreover, it should be considered that when food industry waste material was used for DS production (i.e., the residue of the squeezing for the production of *V. macrocarpon* juices), the anthocyanin profile could be less visible, but still present.

**Figure 1:**
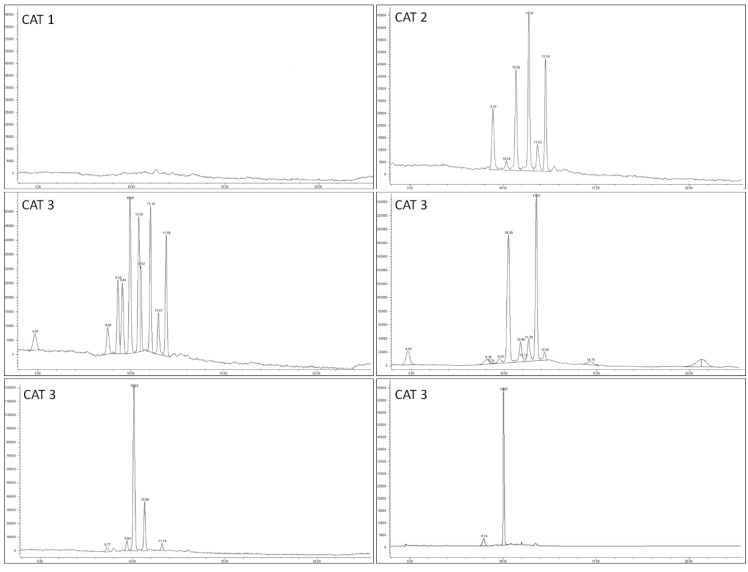
Representative chromatograms of the three main categories into which the 24 *Vaccinium macrocarpon*-based dietary supplements were grouped. CAT 1 groups the products that showed a flat chromatogram, indicating not only the absence of *Vaccinium macrocarpon*, but also of any other fruit containing anthocyanins; CAT 2 groups the products that showed a chromatogram typical of *Vaccinium macrocarpon* fruits; CAT 3 groups the products that contained anthocyanins not characteristic of *Vaccinium macrocarpon*.

Comparing the HPLC-UV/Vis chromatogram of the unknown samples to those of identified *V. macrocarpon* fruits, some important information about the raw material may be obtained [56]. The extracts of the observed 24 *V. macrocarpon*-based supplements were analysed by HPLC-UV/VIS in order to compare the anthocyanin profile with the typical *V. macrocarpon* chromatographic pattern. Based on DS chromatograms, the analysed supplements were gathered in three different groups. Figure 1 reports the representing chromatograms of the three different categories, meanwhile the classification of each DS across the three categories is reported in Table 3. The first category (CAT 1) groups the five DS (#8, #11, #12, #21, and #23) that showed a flat chromatogram (Figure 1, Panel A), thus confirming pH differential method data. The second category (CAT 2) gathers the four DS (#1, #5, #9, and #18) that displayed the same anthocyanin pattern of the *V. macrocarpon* fruits analysed in this experimentation (Figure 1, Panel B). Finally, all the others DS (#2, #3, #4, #6, #7, #10, #13, #14, #15, #16, #17, #19, #20, #22, and #24) displayed anthocyanin patterns different from those of *V. macrocarpon* fruits (CAT 3).

Due to the higher sensitivity of mass-spectrometer with respect to the UV/Vis detector, the UHPLC coupled with high resolution tandem mass spectrometer Orbitrap was employed to analyze all 24 DS. In particular, our analyses aimed to confirm either the absence of anthocyanins in the supplements listed in CAT 1, or the exclusive presence of those characteristic of *V. macrocarpon* fruits in the DS grouped in CAT 2. Finally, the supplements reported in CAT 3 were also analysed in order to confirm the absence of the anthocyanins typical of *V. macrocarpon* fruits. 

The UHPLC-MS/MS data confirmed the HPLC-UV/Vis analysis, on the absence of any anthocyanin in the DS listed in CAT 1. On the other hand, #1, #5, #9, and #18 showed the same anthocyanin profile of *V. macrocarpon* fruits, and did not contain atypical anthocyanins. Finally, UHPLC analysis established the absence of *V. macrocarpon* typical anthocyanins in all the DS listed in CAT 3, except for #20. In this case, UHPLC-MS/MS analysis helped in detecting a low amount of *V. macrocarpon*’s anthocyanins, which was not possible to detect through HPLC-UV/Vis. However, #20, as well as all other supplements listed in CAT 3, showed unexpected chromatographic peaks. UHPLC analysis identified these peaks as delphinidin-rutinoside (#2, #3, #6, #7, 10, #14, #15, #17, #19, #20, #22, and #24), delphinidin-glucoside (#3, #4, #6, #7, #10, #13, #14, #15, #16, #17, #19, #20, and #24), petunidin-glucoside (#10 and #20), and cyanidine-3-O-rutinoside (#20). Even though Oszmiański and colleagues reported that delphinidin-derivates may be present only in small quantities with respect to cranberry-typical anthocyanins [55], the opposite situation is shown in all DS listed in CAT 3, as displayed by the output generated by HPLC-UV/Vis analysis.

## 4. Conclusions

In this paper, we showed how several *V. macrocarpon*-based DS currently on the European market displayed several problems during the formulation and manufacture processes. Indeed, 17 of the 24 DS were shown not to comply with the Pharmacopeia criteria of good preparation. Moreover, 16 of 24 DS claimed in the label to ensure an amount of PACs equal to, or greater than, that shown to be active for the healthcare of the urinary tract. However, only six of them had a tPAC equal to that declared in the label. Finally, the chemical fingerprinting displayed that only five DS contained *V. macrocarpon* extract, meanwhile, the misidentification of the raw material was clear in all the others. Among all the observed 24 *V. macrocarpon*-based DS, only one (**#5**) contemporary complied the criteria of good preparation, respected their uniformity of dosage, and contained *V. macrocarpon* (Table 4).

These data suggested that several *V. macrocarpon*-based DS are present in the European market with insufficient quality controls, and that they are frequently mislabeled. Considering that in most cases DS are self-prescribed, and that the consumer relies exclusively on the claim and label of these products, the lack of an appropriate control quality confuses the consumer in choosing a product that should contribute to his personal well-being.

## Figures and Tables

**Table 1:** Mass uniformity test of the 24 *Vaccinium macrocarpon*-based dietary supplements. The pharmaceutical forms (FF) of each package were evaluated following three different criteria. Criteria 1: FF complied with the test if not more than one individual mass was outside of the percentage established of the average mass (5% for tablets and 7,5% for capsules and sachets); Criteria 2: FF failed to comply with the test if one individual mass was outside of the double of these limits (10% for tablets and 15% for capsules and sachets); Criteria 3: FF failed to comply with the test if the ratio between the claimed mass and the average mass exceeded the 2%. The column “%CV” reports the percentage difference of the measured weight compared to that declared in each package.

#	Weight (g)		%CV	Criteria 1	Criteria 2	Criteria 3
	Claimed	Average of Each FF				
1	3.00	2.9776 ± 0.0326	99.25%	yes	yes	yes
2	4.50	4.5126 ± 0.0473	100.28%	yes	yes	yes
3	4.00	3.9816 ± 0.0209	99.54%	yes	yes	yes
4	4.15	4.2152 ± 0.0332	101.57%	yes	yes	yes
5	5.00	5.0545 ± 0.0318	101.09%	yes	yes	yes
6	3.30	3.3309 ± 0.1732	100.93%	yes	yes	yes
7	3.50	3.4611 ± 0.0926	98.88%	yes	yes	yes
8	2.00	1.8799 ± 0.2519	93.99%	no	no	yes
9	3.50	4.1581 ± 0.6992	118.80%	no	no	no
10	0.90	0.3455 ± 0.0201	38.38%	no	no	no
11	0.35	0.3971 ± 0.0091	115.10%	yes	yes	no
12	0.53	0.5753 ± 0.0833	107.93%	no	no	yes
13	0.42	0.4392 ± 0.0131	104.07%	yes	yes	yes
14	0.50	0.5011 ± 0.0113	100.22%	yes	yes	yes
15	20.00	23.192 ± 0.5796	115.96%	yes	yes	no
16	1.20	1.1901 ± 0.0144	99.17%	no	yes	yes
17	1.20	1.0260 ± 0.0142	85.50%	no	yes	no
18	0.75	0.7478 ± 0.0063	99.70%	yes	yes	yes
19	0.55	0.5362 ± 0.0058	97.49%	yes	yes	yes
20	1.03	1.0554 ± 0.0052	102.46%	yes	yes	yes
21	0.97	0.9722 ± 0.0113	100.43%	yes	yes	yes
22	0.68	0.6901 ± 0.0194	102.23%	no	yes	yes
23	0.88	0.8747 ± 0.0093	99.96%	yes	yes	yes
24	1.15	1.1972 ± 0.0111	104.37%	yes	yes	yes

**Table 2:** Quantification of total proanthocyanidins content (tPACs) in the 24 *Vaccinium macrocarpon*-based dietary supplements by BL-DMAC assay. The column “method” reports the claimed analytical method used by the manufacturer for the quantification of tPACs. Results are expressed as mean of three different replicates ± SD, using external calibration curves of dimer PAC-A type. In the column “%CV”, the percentage differences of tPACs between the value declared and that calculated via BL-DMAC assay are reported for each product.

#		mg PAC per FF		%CV
	Claimed	Method	Real	
1	36		5.54 ± 0.27	15.39%
2	n.d.		7.45 ± 0.45	
3	43.2		41.66 ± 1.13	96.44%
4	36	BL-DMAC	34.86 ± 1.73	96.83%
5	18	BL-DMAC	17.84 ± 1.34	99.11%
6	36	BL-DMAC	4.91 ± 0.22	13.64%
7	50		4.41 ± 0.01	8.82%
8	18	BL-DMAC	2.48 ± 0.05	13.78%
9	45	BL-DMAC	37.89 ± 1.59	84.20%
10	46.8	BL-DMAC	0.55 ± 0.05	1.18%
11	n.d.		1.51 ± 0.09	
12	n.d.		2.79 ± 0.14	
13	n.d.		3.68 ± 0.19	
14	30		6.01 ± 0.23	20.03%
15	n.d.		1.13 ± 0.06	
16	18		0.93 ± 0.03	5.17%
17	36		5.31 ± 0.44	14.75%
18	20		6.48 ± 0.21	32.40%
19	14.4	BL-DMAC	13.92 ± 0.28	96.67%
20	54	BL-DMAC	32.91 ± 0.91	60.94%
21	n.d.		0.38 ± 0.02	
22	n.d.		17.12 ± 0.63	
23	n.d.		0.28 ± 0.02	
24	36	BL-DMAC	37.17 ± 1.53	103.25%

**Table 3:** Quantification of total anthocyanin content (TAC) and chemical fingerprinting of the 24 *Vaccinium macrocarpon*-based dietary supplements. Results are the mean of three different replicates, and they are expressed as mg of cyanidine chloride equivalent ± SD. The different supplements are grouped in three different categories in the column “CAT”, depending on the different chromatogram recorded at λ = 520 nm (see Figure 1). The non-typical anthocyanins found in each sample are reported in the column “UHPLC-Orbitrap”. (D3Ru = delphinidin-rutinoside; D3Gl = delphinidin-glucoside; Pet3Gl = petunidin-glucoside; C3Ru = cyanidine-3-rutinoside).

#	TAC per FF	Chemical Fingerprinting						
		HPLC-UV/Vis			UHPLC-Orbitrap			
		CAT 1	CAT 2	CAT 3	D3Ru	D3Gl	Pet3Gl	C3Ru
1	3.601 ± 0.211		x					
2	21.997 ± 1.713			x	x	x		
3	5.462 ± 0.343			x	x	x		
4	13.317 ± 0.258			x				
5	8.925 ± 0.241		x					
6	2.322 ± 0.131			x	x	x		
7	0.389 ± 0.032			x	x	x		
8	n.d.	x						
9	19.501 ± 1.569		x					
10	4.859 ± 0.121			x	x	x	x	
11	n.d.	x						
12	n.d.	x						
13	6.325 ± 0.589			x				
14	0.444 ± 0.021			x	x	x		
15	4.359 ± 0.356			x	x	x		
16	2.134 ± 0.123			x				
17	1.134 ± 0.0458			x	x	x		
18	3.184 ± 0.192		x					
19	1.102 ± 0.0874			x	x	x		
20	6.439 ± 0.255			x	x	x	x	
21	n.d.	x						
22	2.907 ± 0.287			x	x			
23	n.d.	x						
24	3.361 ± 0.055			x	x	x		x

**Table 4:** Summary table of the quality control tests carried out on 24 *Vaccinium macrocarpon*-based dietary supplements. The symbol “✓” marks the passing of the relative test for each product. On the contrary, the symbol “x” marks when the DS failed to comply with the test. Criteria 1: FF complied with the test if not more than one individual mass was outside of the percentage established of the average mass (5% for tablets and 7.5% for capsules and sachets); Criteria 2: FF failed to comply with the test if one individual mass was outside of the double of these limits (10% for tablets and 15% for capsules and sachets); Criteria 3: FF failed to comply with the test if the ratio between the claimed mass and the average mass exceeded the 2%. The column “36 mg/day” reports if, following the suggested posology, the administration of at least 36 mg of PACs was guaranteed, while the adjacent column reports if the dosage claimed in the label was correct. The other columns respectively report the detection of anthocyanins (TAC), and whether the anthocyanin pattern was different from those of the *V. macrocarpon* fruits analysed in this experimentation (FP).

	Uniformity of Mass			PACs Content		Cranberry	
#	Criteria 1	Criteria 2	Criteria 3	36 mg/day	Correct Dosage	TAC	FP
1	✓	✓	✓	x	x	✓	✓
2	✓	✓	✓	x	n.r.	✓	x
3	✓	✓	✓	✓	✓	✓	x
4	✓	✓	✓	✓	✓	✓	x
5	✓	✓	✓	✓	✓	✓	✓
6	✓	✓	✓	x	x	✓	x
7	✓	✓	✓	x	x	✓	x
8	x	x	✓	x	x	x	x
9	x	x	x	✓	x	✓	✓
10	x	x	x	x	x	✓	x
11	✓	✓	x	x	n.r.	x	x
12	x	x	✓	x	n.r.	x	x
13	✓	✓	✓	x	n.r.	✓	x
14	✓	✓	✓	x	x	✓	x
15	✓	✓	x	x	n.r.	✓	x
16	x	✓	✓	x	x	✓	x
17	x	✓	x	x	x	✓	x
18	✓	✓	✓	x	x	✓	✓
19	✓	✓	✓	✓	✓	✓	x
20	✓	✓	✓	x	x	✓	✓
21	✓	✓	✓	x	n.r.	x	x
22	x	✓	✓	✓	n.r.	✓	x
23	✓	✓	✓	x	n.r.	x	x
24	✓	✓	✓	✓	✓	✓	x

n.r. = PACs was not reported in the label of DS

## Supplementary Materials

The following are available online at https://www.mdpi.com/2072-6643/12/4/992/s1, Table S1: Inclusion mass list of anthocyanins that are not characteristic of *Vaccinium macrocarpon* fruits and that were researched in the 24 cranberry-based dietary supplements. Figure S1–S24: HPLC-UV/Vis analysis of the 24 cranberry-based dietary supplements.
