# Supplementary material for: Vaccinium macrocarpon (Cranberry)-Based Dietary Supplements: Variation in Mass Uniformity, Proanthocyanidin Dosage and Anthocyanin Profile Demonstrates Quality Control Standard Needed
Source: Nutrients. 2020 Apr 3;12(4):992. doi: 10.3390/nu12040992 (PMC7230672; doi:10.3390/nu12040992)
Supplement: Supplementary file 1 [file nutrients-12-00992-s001.zip › Supporting Table 1.docx]

**Supporting Table 1**: Inclusion masses list of anthocyanin that are not characteristic in *Vaccinium macrocarpon* fruits and that were researched in the 24 cranberry-based dietary supplements.

| **Mass (m/z)** | **Formula** | **Polarity** | **Common Name** |
| --- | --- | --- | --- |
| 419,09727 | C_20_H_18_O_10_ | Positive | Cyanidin-3-O-Arabinoside |
| 433,11292 | C_21_H_20_O_10_ | Positive | Pelargonidin-3-O-Arabinoside |
| 433,11292 | C_21_H_20_O_10_ | Positive | Peonidin-3-O-Arabinoside |
| 449,10784 | C_21_H_20_O_11_ | Positive | Cyanidin-3-O-Galactoside |
| 449,10784 | C_21_H_20_O_11_ | Positive | Cyanidin-3-O-Glucoside |
| 463,12349 | C_22_H_22_O_11_ | Positive | Peonidin-3-O-Galactoside |
| 465,10275 | C_21_H_20_O_12_ | Positive | Delphinidin-3-O-Glucoside |
| 535,10823 | C_24_H_22_O_14_ | Positive | Cyanidin-3-(6'-malonylglucoside) |
| 579,17083 | C_27_H_30_O_14_ | Positive | Pelargonidin-3-O-Rutinoside |
| 593,07733 | C_25_H_20_O_17_ | Positive | Cyanidin 3-Dioxaloylglucoside |
| 595,16575 | C_27_H_30_O_15_ | Positive | Cyanidin-3-O-Rutinoside |
| 595,16575 | C_27_H_30_O_15_ | Positive | Pelargonidin-3,5-diglucoside |
| 627,15558 | C_27_H_30_O_17_ | Positive | Delphinidin-3,5-diglucoside |
| 611,16066 | C_27_H_30_O_16_ | Positive | Cyanidin-3,5-diglucoside |
