# Supplementary figures and images for: Vaccinium macrocarpon (Cranberry)-Based Dietary Supplements: Variation in Mass Uniformity, Proanthocyanidin Dosage and Anthocyanin Profile Demonstrates Quality Control Standard Needed
Source: Nutrients. 2020 Apr 3;12(4):992. doi: 10.3390/nu12040992 (PMC7230672; doi:10.3390/nu12040992)

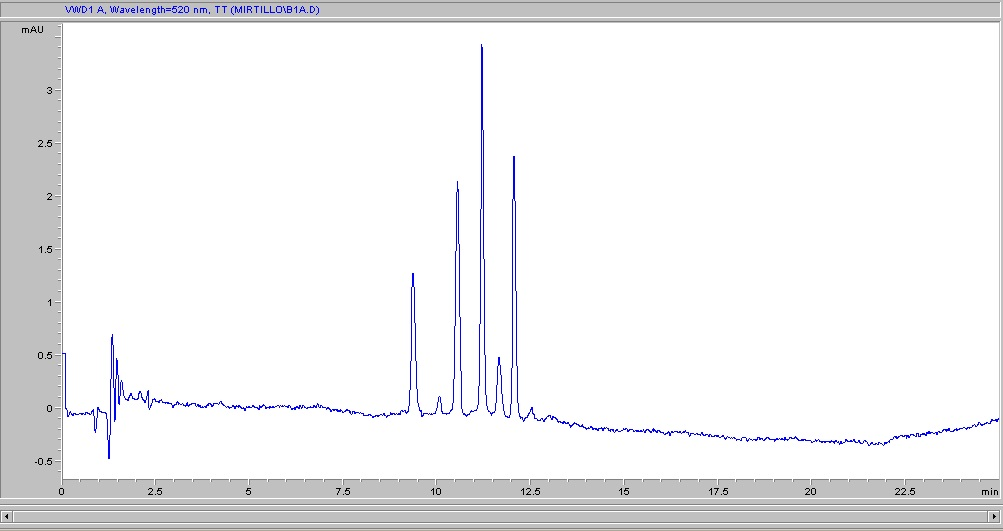

Supplement: Supplementary file 1 [file nutrients-12-00992-s001.zip › Supplementary Figure 1-24/Supplementary Picture 1.tif]

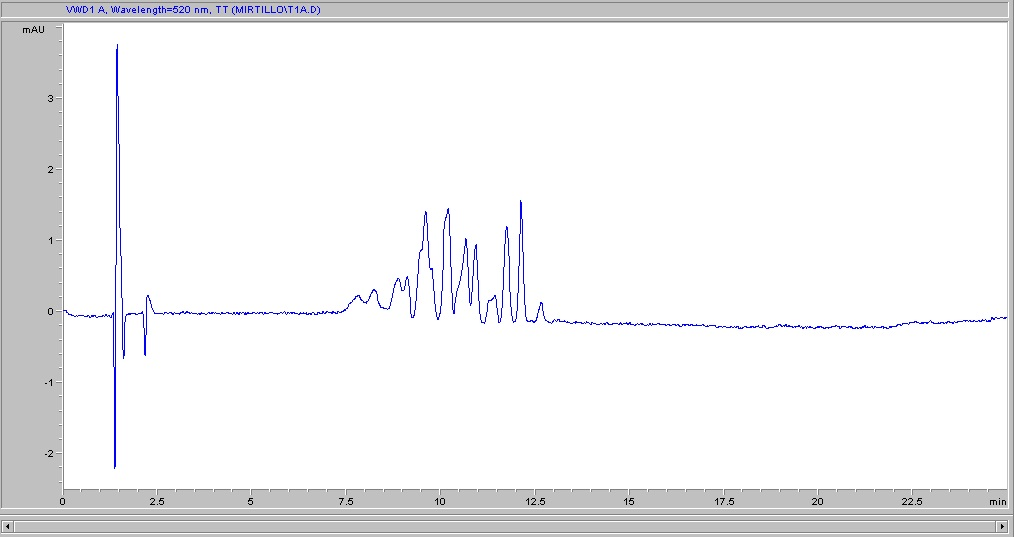

Supplement: Supplementary file 1 [file nutrients-12-00992-s001.zip › Supplementary Figure 1-24/Supplementary Picture 10.tif]

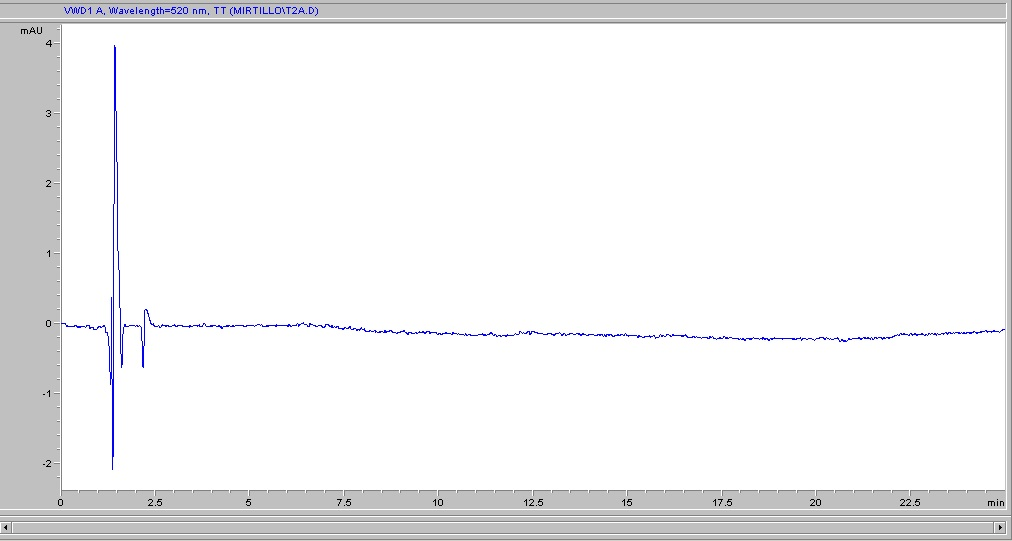

Supplement: Supplementary file 1 [file nutrients-12-00992-s001.zip › Supplementary Figure 1-24/Supplementary Picture 11.tif]

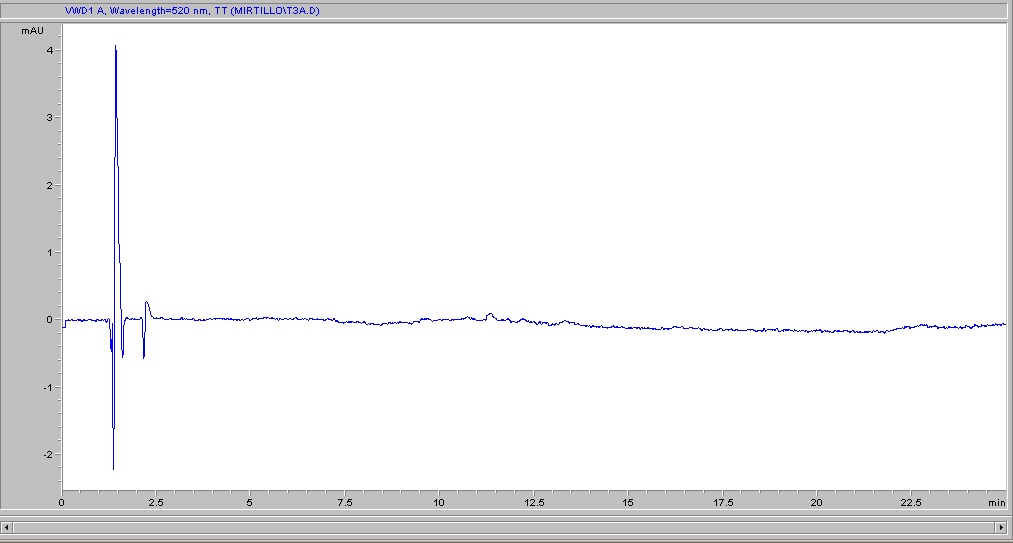

Supplement: Supplementary file 1 [file nutrients-12-00992-s001.zip › Supplementary Figure 1-24/Supplementary Picture 12.tif]

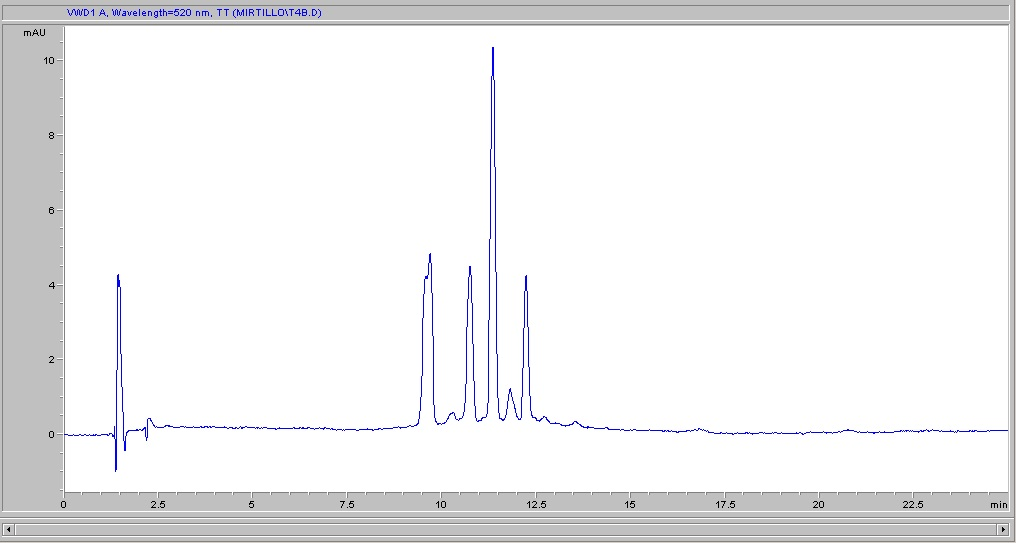

Supplement: Supplementary file 1 [file nutrients-12-00992-s001.zip › Supplementary Figure 1-24/Supplementary Picture 13.tif]

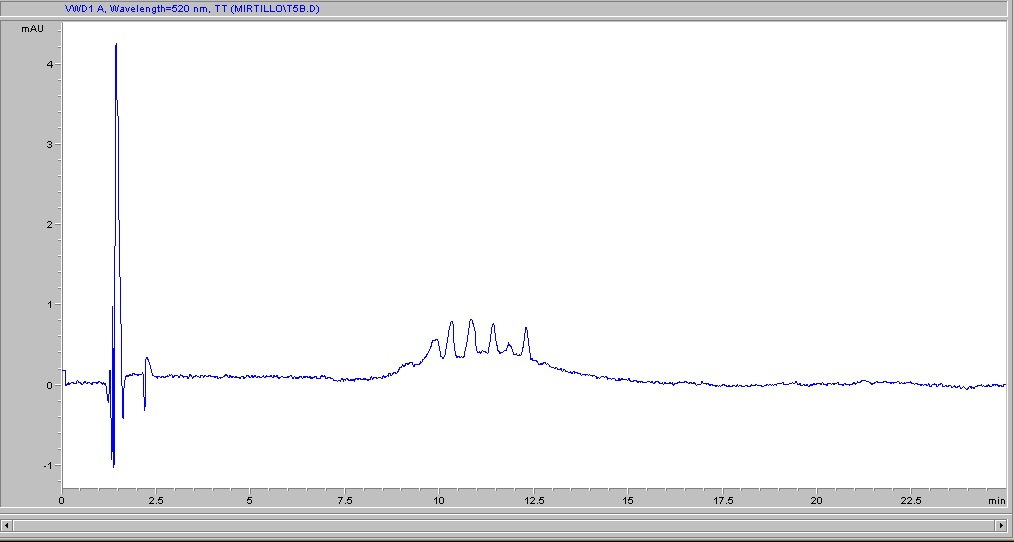

Supplement: Supplementary file 1 [file nutrients-12-00992-s001.zip › Supplementary Figure 1-24/Supplementary Picture 14.tif]

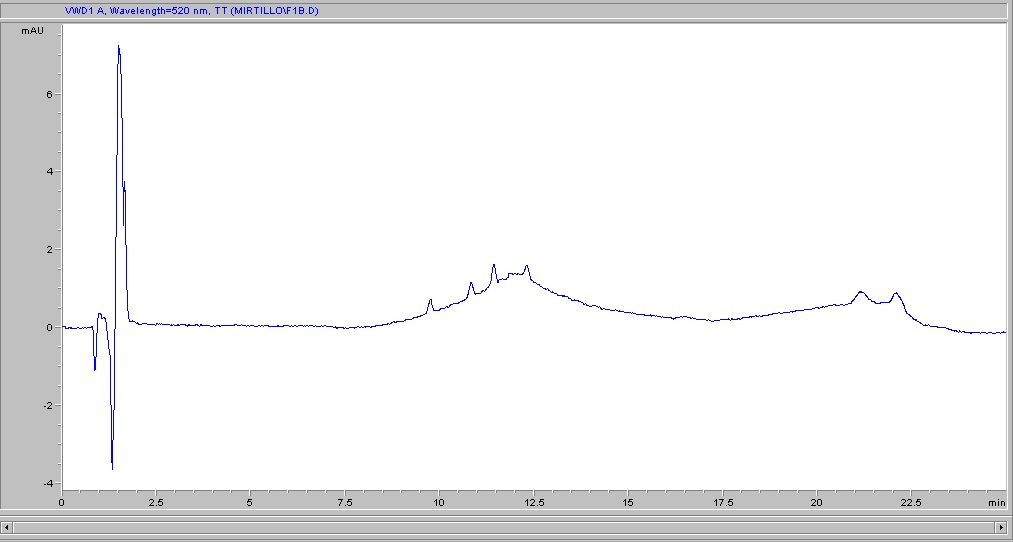

Supplement: Supplementary file 1 [file nutrients-12-00992-s001.zip › Supplementary Figure 1-24/Supplementary Picture 15.tif]

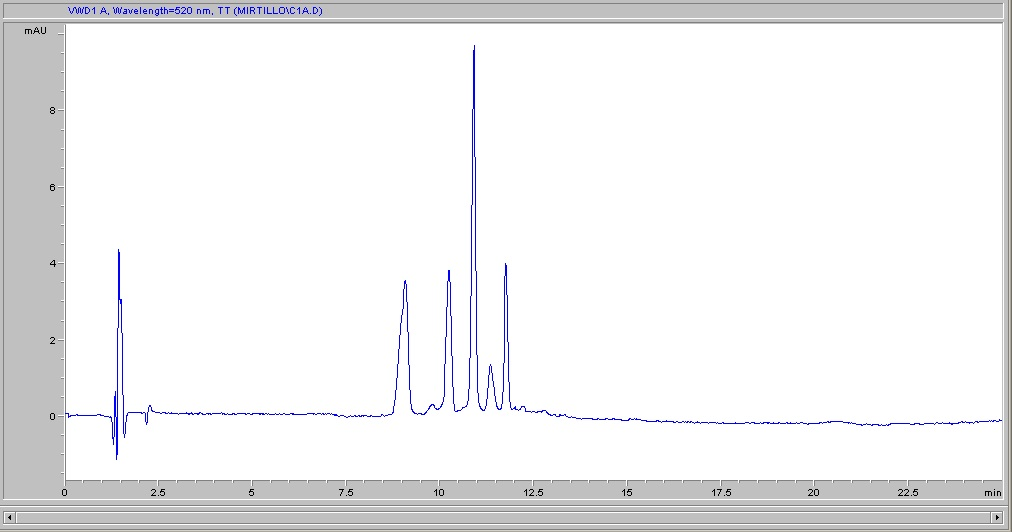

Supplement: Supplementary file 1 [file nutrients-12-00992-s001.zip › Supplementary Figure 1-24/Supplementary Picture 16.tif]

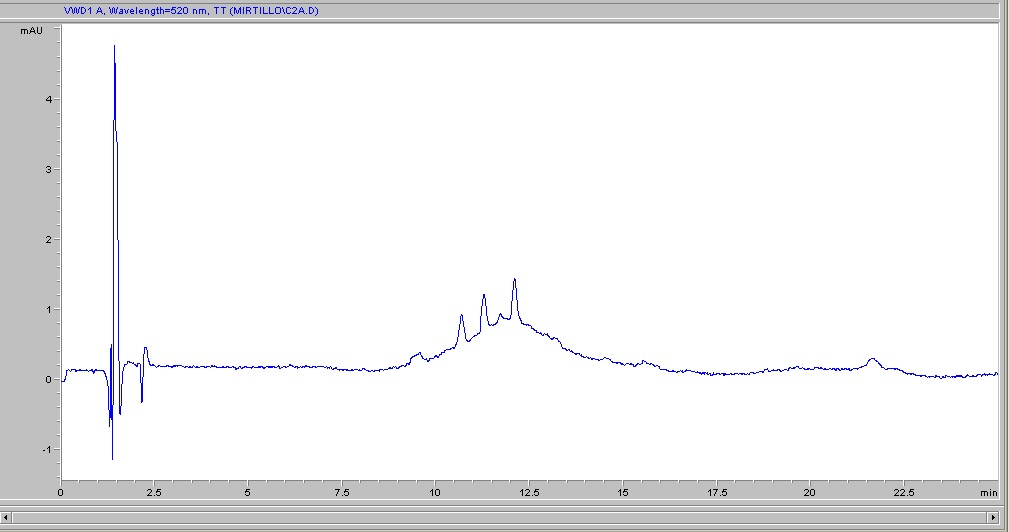

Supplement: Supplementary file 1 [file nutrients-12-00992-s001.zip › Supplementary Figure 1-24/Supplementary Picture 17.tif]

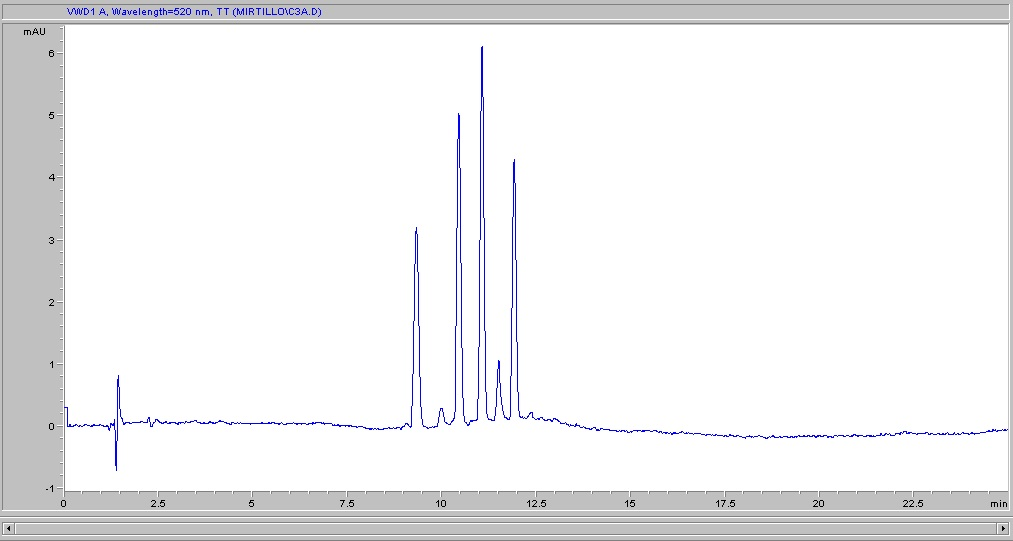

Supplement: Supplementary file 1 [file nutrients-12-00992-s001.zip › Supplementary Figure 1-24/Supplementary Picture 18.tif]

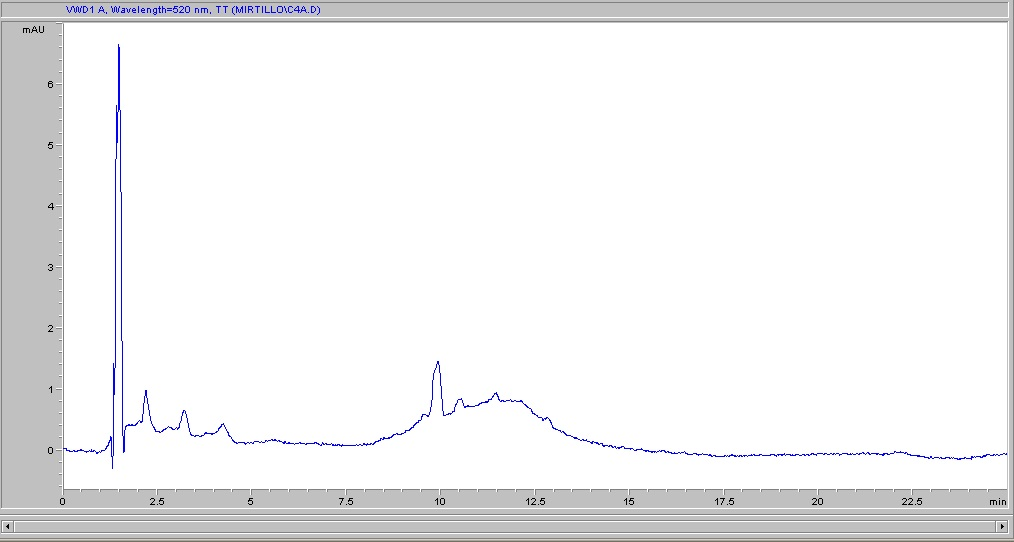

Supplement: Supplementary file 1 [file nutrients-12-00992-s001.zip › Supplementary Figure 1-24/Supplementary Picture 19.tif]

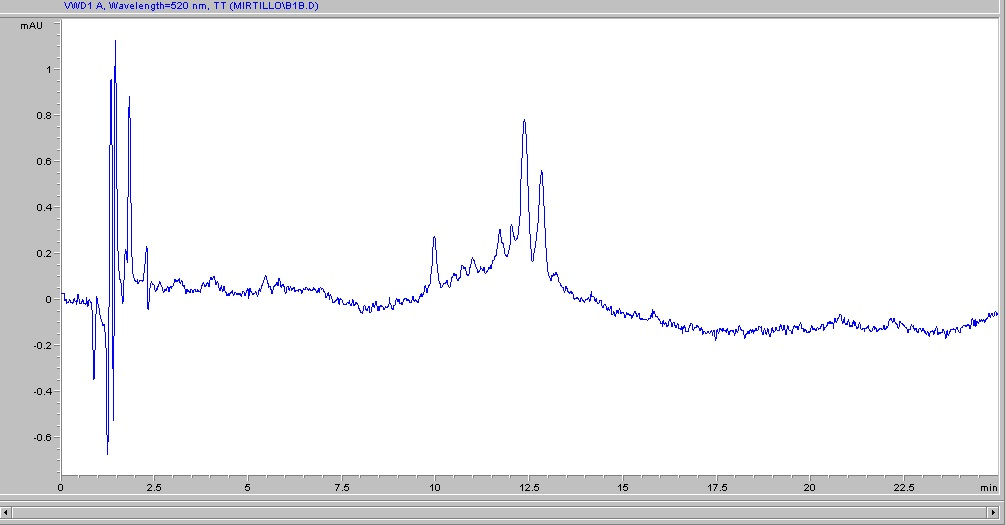

Supplement: Supplementary file 1 [file nutrients-12-00992-s001.zip › Supplementary Figure 1-24/Supplementary Picture 2.tif]

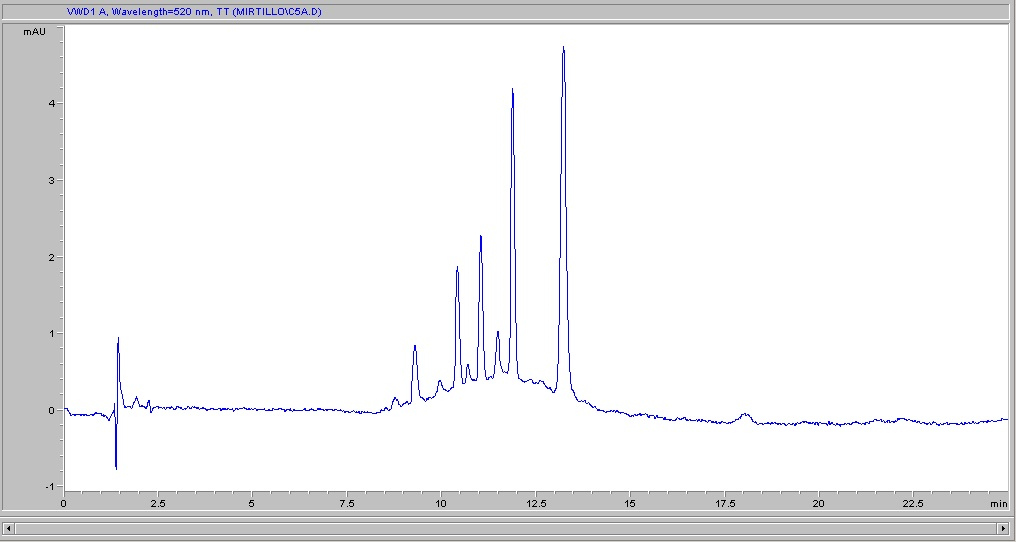

Supplement: Supplementary file 1 [file nutrients-12-00992-s001.zip › Supplementary Figure 1-24/Supplementary Picture 20.tif]

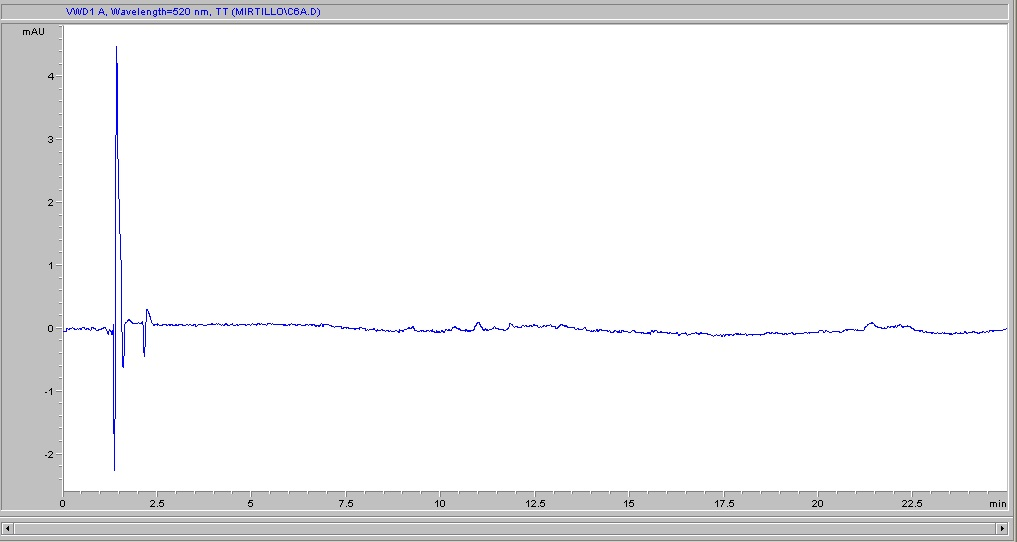

Supplement: Supplementary file 1 [file nutrients-12-00992-s001.zip › Supplementary Figure 1-24/Supplementary Picture 21.tif]

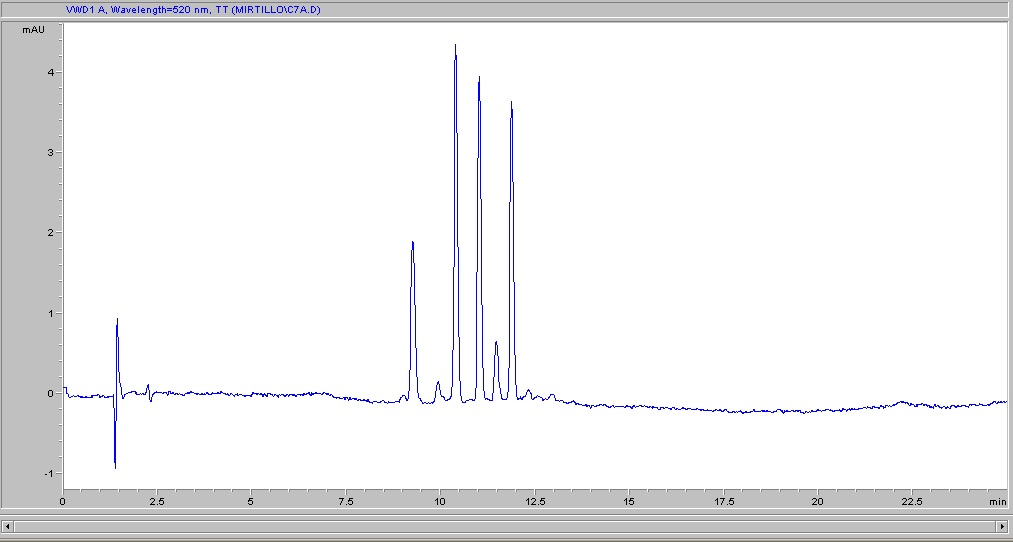

Supplement: Supplementary file 1 [file nutrients-12-00992-s001.zip › Supplementary Figure 1-24/Supplementary Picture 22.tif]

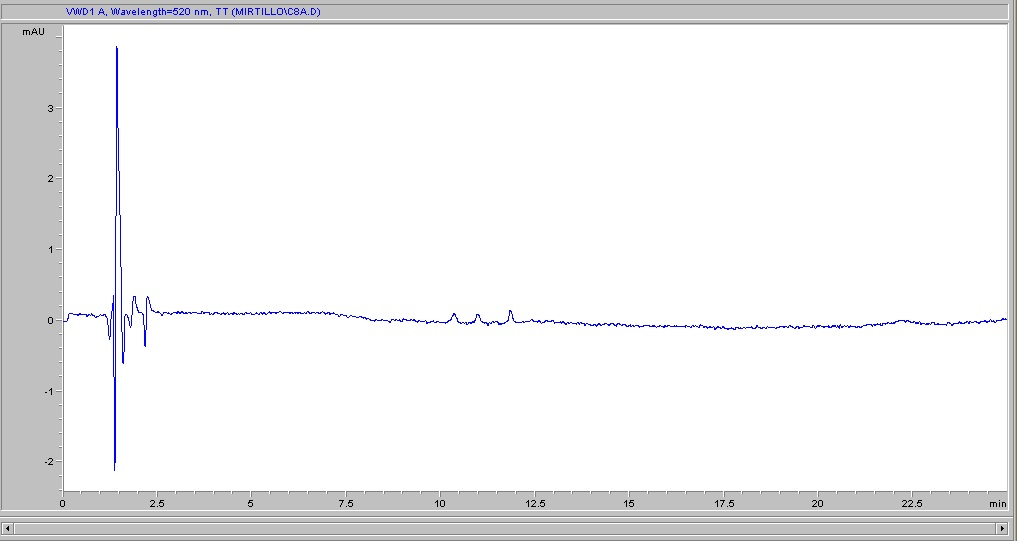

Supplement: Supplementary file 1 [file nutrients-12-00992-s001.zip › Supplementary Figure 1-24/Supplementary Picture 23.tif]

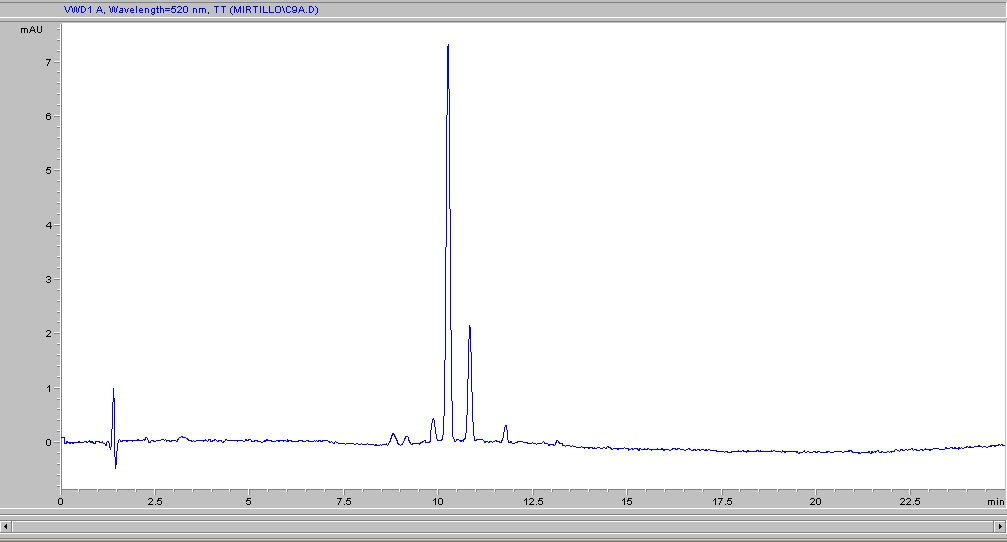

Supplement: Supplementary file 1 [file nutrients-12-00992-s001.zip › Supplementary Figure 1-24/Supplementary Picture 24.tif]

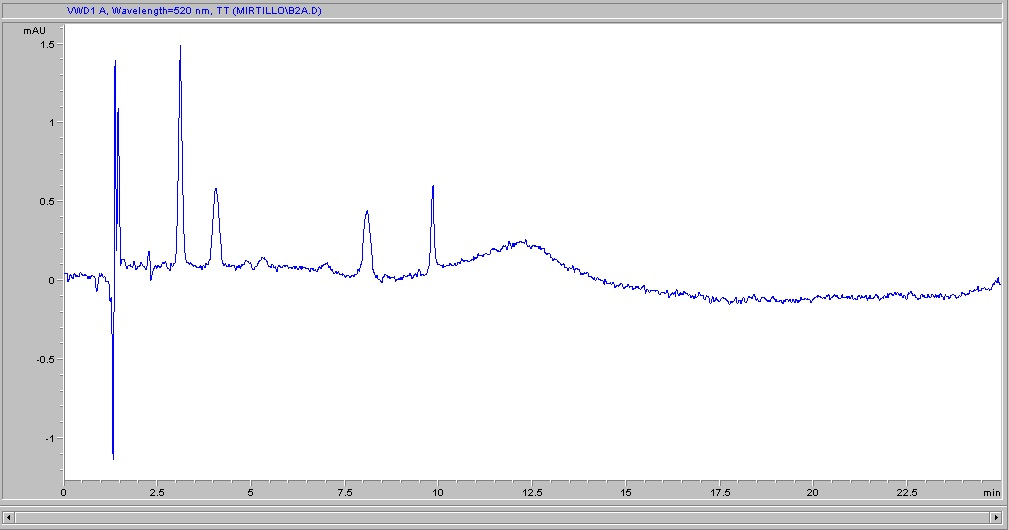

Supplement: Supplementary file 1 [file nutrients-12-00992-s001.zip › Supplementary Figure 1-24/Supplementary Picture 3.tif]

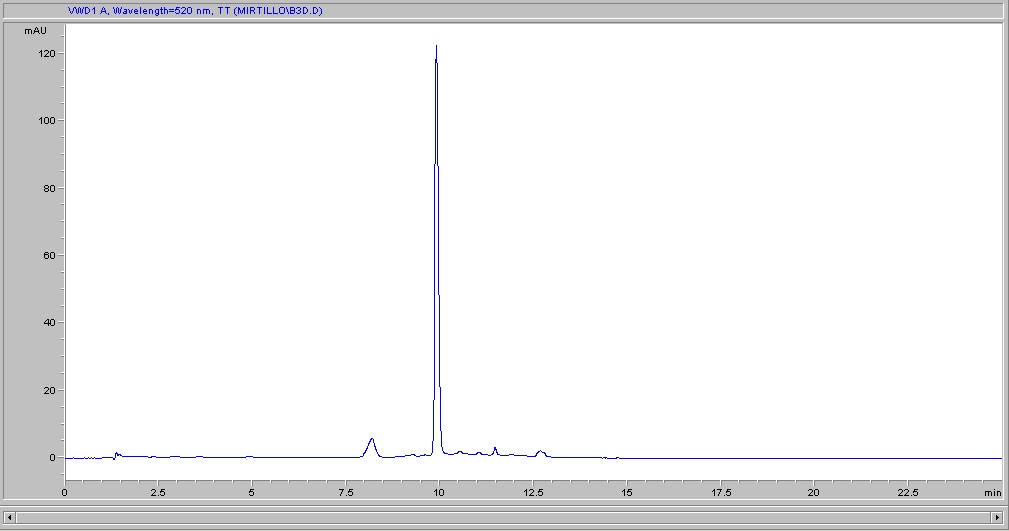

Supplement: Supplementary file 1 [file nutrients-12-00992-s001.zip › Supplementary Figure 1-24/Supplementary Picture 4.tif]

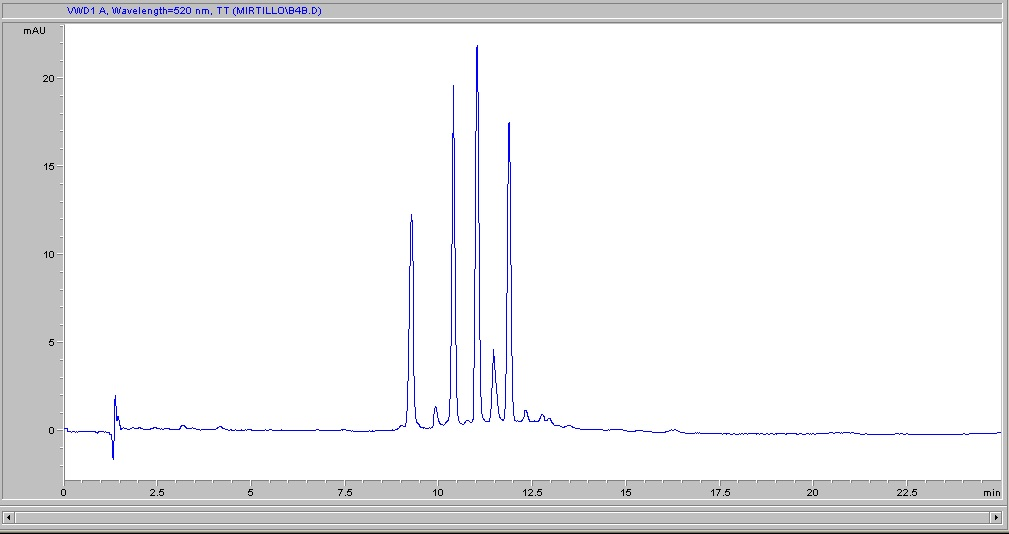

Supplement: Supplementary file 1 [file nutrients-12-00992-s001.zip › Supplementary Figure 1-24/Supplementary Picture 5.tif]

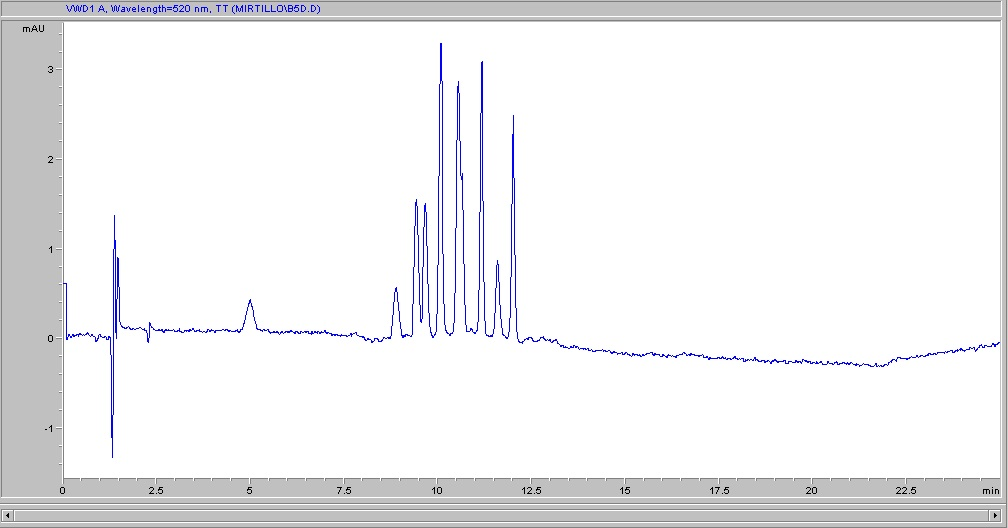

Supplement: Supplementary file 1 [file nutrients-12-00992-s001.zip › Supplementary Figure 1-24/Supplementary Picture 6.tif]

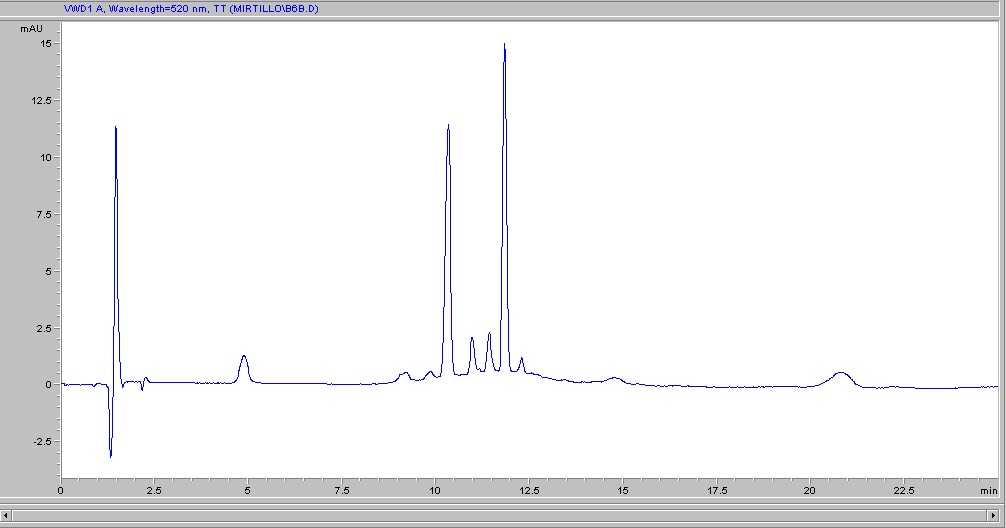

Supplement: Supplementary file 1 [file nutrients-12-00992-s001.zip › Supplementary Figure 1-24/Supplementary Picture 7.tif]

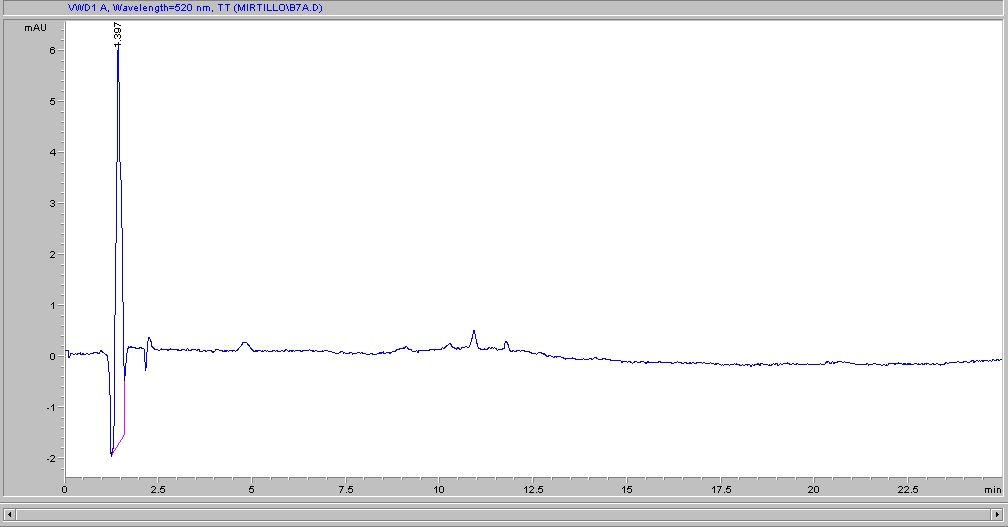

Supplement: Supplementary file 1 [file nutrients-12-00992-s001.zip › Supplementary Figure 1-24/Supplementary Picture 8.tif]

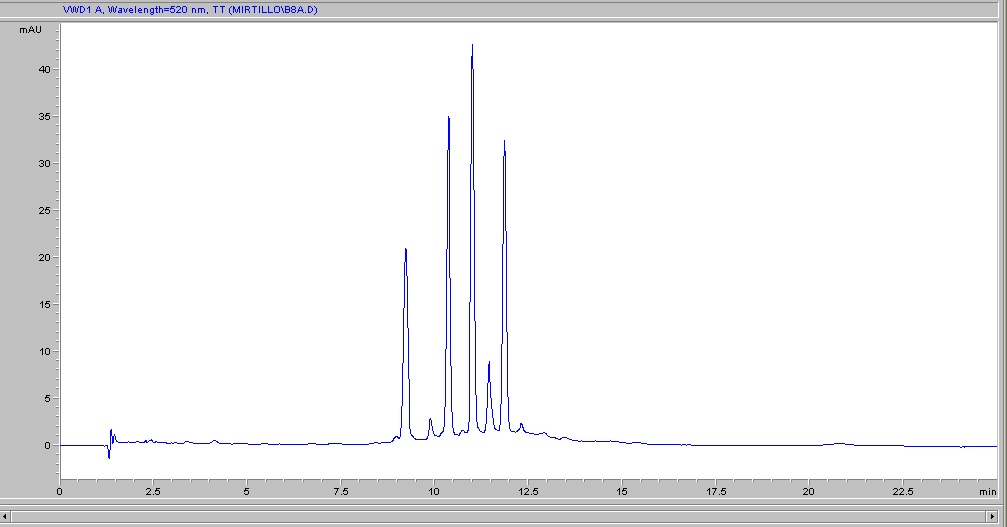

Supplement: Supplementary file 1 [file nutrients-12-00992-s001.zip › Supplementary Figure 1-24/Supplementary Picture 9.tif]
